# Intervertebral disc distraction stiffness predicts endplate subsidence following transforaminal interbody cage expansion: an ex vivo study

**DOI:** 10.1007/s00586-025-09715-x

**Published:** 2026-01-09

**Authors:** Kay A. Raftery, Hannah Levy, Rananjay Singh, Mohammed Madi, Thomas D. Slater, Antony J. Crossman, Angela E. Kedgley, Brett A. Freedman, Nicolas Newell

**Affiliations:** 1 Department of Bioengineering, Imperial College London, London, UK; 2 Department of Orthopaedic Surgery, Mayo Clinic, Rochester, USA; 3 Faculty of Medicine, Imperial College London, London, UK; 4 Royal National Orthopaedic Hospital NHS Trust, Stanmore, UK

**Keywords:** Lumbar interbody fusion, Expandable cage, Subsidence, IVD, Torque, Distraction

## Abstract

**Purpose:**

Expandable cages have the potential to mitigate the currently high subsidence rates following transforaminal lumbar interbody fusion (TLIF), but are liable to over-distraction *in situ.* This may be due to the undefined patient-specific expansion threshold of the intervertebral disc (IVD) space. This study aimed to elucidate whether IVD properties affect the torque required to expand the cage within the IVD space, and determine the association between achieved torque, distraction stiffness, and subsidence severity.

**Methods:**

Fifteen cadaveric L3-L4 and L4-L5 samples were prepared with the TLIF approach. Under 100 N compression, the torque required to expand the cage per half-turn, alongside the changes to IVD and cage height, were recorded until maximum cage expansion. Subsidence depth was measured after subsequent cyclic loading, and the surface area of removed IVD tissue was quantified post-test.

**Results:**

Peak torque was inversely associated with preloaded IVD height (B: –0.34, *p* < 0.001) and the percentage of IVD removed (B: –0.04, *p* < 0.01). IVD distraction stiffness was associated with preloaded IVD height only (B: -0.19, *p* < 0.001). There was no association with IVD or facet degeneration. When subsidence depth was normalised to bone mineral density, a positive correlation was observed with peak torque and cage expansion stiffness (both *p* < 0.05).

**Conclusion:**

The torque required to expand interbody cages in situ is relevant to subsidence risk, and depends on IVD geometry and the amount of residual tissue. Thus, short IVDs should be thoroughly prepared to alleviate excessive stiffness during cage expansion.

**Supplementary Information:**

The online version contains supplementary material available at 10.1007/s00586-025-09715-x.

## Introduction

Lumbar interbody fusion surgery aims to treat a range of spinal degenerative conditions associated with low back pain. Transforaminal lumbar interbody fusion (TLIF) presents benefits such as operative efficiency and reduced invasiveness, yet patients are three times more likely to sustain post-operative subsidence relative to other approaches [1].

Expandable interbody cages are a promising solution to mitigate the disadvantages of TLIF with static interbody cages, offering in situ distraction for improved sagittal alignment in minimally invasive circumstances [2]. However, in practice, the post-operative subsidence risk using expandable cages is 2.1 times higher than static [3], attributed to cage over-expansion [4]. Surgeons are advised to utilise haptic feedback to avoid this undesirable outcome, yet it is recognised that achieving accurate or reproducible torque magnitudes between patients is challenging, further hindered by the required learning curve [5].

Currently, the relationship between distraction torque and subsidence is not well established. Previously reported torque-to-subsidence values are highly variable between specimens (2.4–3.9 Nm) [6], and have been shown to be independent of the forces at the cage-endplate interface [7]. Moreover, both aforementioned studies were based on ex vivo simulations using vertebral replacement cages, which neglected the effect of soft tissue tension from the intervertebral disc (IVD).

Thorough discectomy during TLIF is challenging, with the amount of tissue removed ranging from 83% on the ipsilateral side to as little as 50% on the contralateral side [8]. Whilst tension of the remaining annulus fibrosus (AF) and surrounding ligaments may promote post-operative segmental stability [9], excessive tension could exacerbate compressive force at the endplate-cage interface, encouraging subsidence. Additionally, whilst the effects of IVD degeneration on the tensile behaviour of AF lamellae have been extensively characterised [10], it is unknown how IVD degeneration in its structural form contributes to the distraction stiffness of the IVD space.

Factors contributing to IVD distraction stiffness and their relation to post-operative subsidence have not been investigated previously. A better understanding of these relationships may enable the definition of patient-specific torque thresholds that allow sagittal lordosis to be optimised whilst minimising over-distraction risk.

The aims of this cadaveric study were to:

i) Characterise the variability in torque-distraction profiles of the lumbar IVD space during cage expansion;
ii) Describe the relationship between peak torque achieved and distraction stiffness with subsidence depth following cyclic testing;
iii) Determine which morphological and geometric properties of the IVD can predict distraction stiffness.

## Methods

### Specimen Preparation

After obtaining ethical approval from the Imperial College Tissue Bank Ethics Committee (approval number: 22/WA/0214), 15 half vertebra-IVD-half vertebra samples were harvested from 11 cadaveric lumbar spines (64 ± 5 years) (Table 1). L3-L4 and L4-L5 IVDs were used as they represent the most operated levels for TLIF [11]. Lumbar spines were dissected, removing all surrounding soft tissue and the interspinous, supraspinous, and intertransverse ligaments. The posterior longitudinal ligament and the ligamentum flavum were partially resected for IVD access, and the anterior longitudinal and facet capsular ligaments were retained.

A consultant spinal surgeon (MM) performed an *en bloc* facetectomy on the left facet using an osteotome and high-speed drill (Midas Rex™, Medtronic). A box annulotomy and discectomy were performed with a scalpel and pituitary rongeurs, respectively. Both endplates were prepared using straight and curved curettes (Medtronic) through the transforaminal approach, until the complete removal of the cartilaginous endplate. This was confirmed under tactile feedback on both the cranial and caudal endplates. The number of passes was dependent on the IVD space size and volume of IVD remaining at each level.

**Table 1:** Donor demographic information. samples are numbered “i.j.” where i corresponds to the donor, and j corresponds to the vertebral level. Note that endplate volumetric BMD (vBMD) was not calculated for sample #7 which fractured during the TLIF surgery (indicated with an asterisk). Trabecular vBMD was used for osteoporosis classification (healthy: vBMD > 120 mg/cm^3^; osteopoenic: 80 mg/cm^3^ ≤ vBMD ≤ 120 mg/cm^3^; osteoporotic: vBMD < 80 mg/cm^3^)

Donor (#)	Gender	Age (years)	Level	Trabecular vBMD (mg/cm^3^)	Endplate vBMD (mg/cm^3^)	Osteoporosis classification	Pfirrmann grade	Facet degeneration score	T2 relaxation time (ms)
1	M	64	L3-L4	115.03	245.92	Osteopoenic	3	0	58.80
2.1	M	68	L3-L4	110.40	206.30	Osteopoenic	4	3	53.71
2.2	M	68	L4-L5	115.71	230.45	Osteopoenic	4	3	56.10
3	M	61	L4-L5	55.92	139.47	Osteoporotic	3	2	56.72
4	M	70	L4-L5	116.65	277.07	Osteopoenic	4	3	50.05
5	M	61	L4-L5	146.61	379.39	Healthy	2	0	61.27
6	F	57	L3-L4	129.85	264.55	Healthy	2	1	67.25
7*	M	72	L4-L5	124.60	-	Healthy	5	3	63.93
8	F	67	L4-L5	81.38	158.77	Osteopoenic	3	2	60.90
9.1	F	65	L3-L4	171.94	346.89	Healthy	3	2	57.73
9.2	F	65	L4-L5	188.26	339.09	Healthy	4	3	54.56
10.1	F	62	L3-L4	88.14	192.04	Osteopoenic	2	1	69.00
10.2	F	62	L4-L5	92.83	187.51	Osteopoenic	3	3	58.00
11.1	F	59	L3-L4	57.05	221.98	Osteoporotic	3	2	54.01
11.2	F	59	L4-L5	58.68	177.74	Osteoporotic	3	2	55.29

**Fig. 1:**
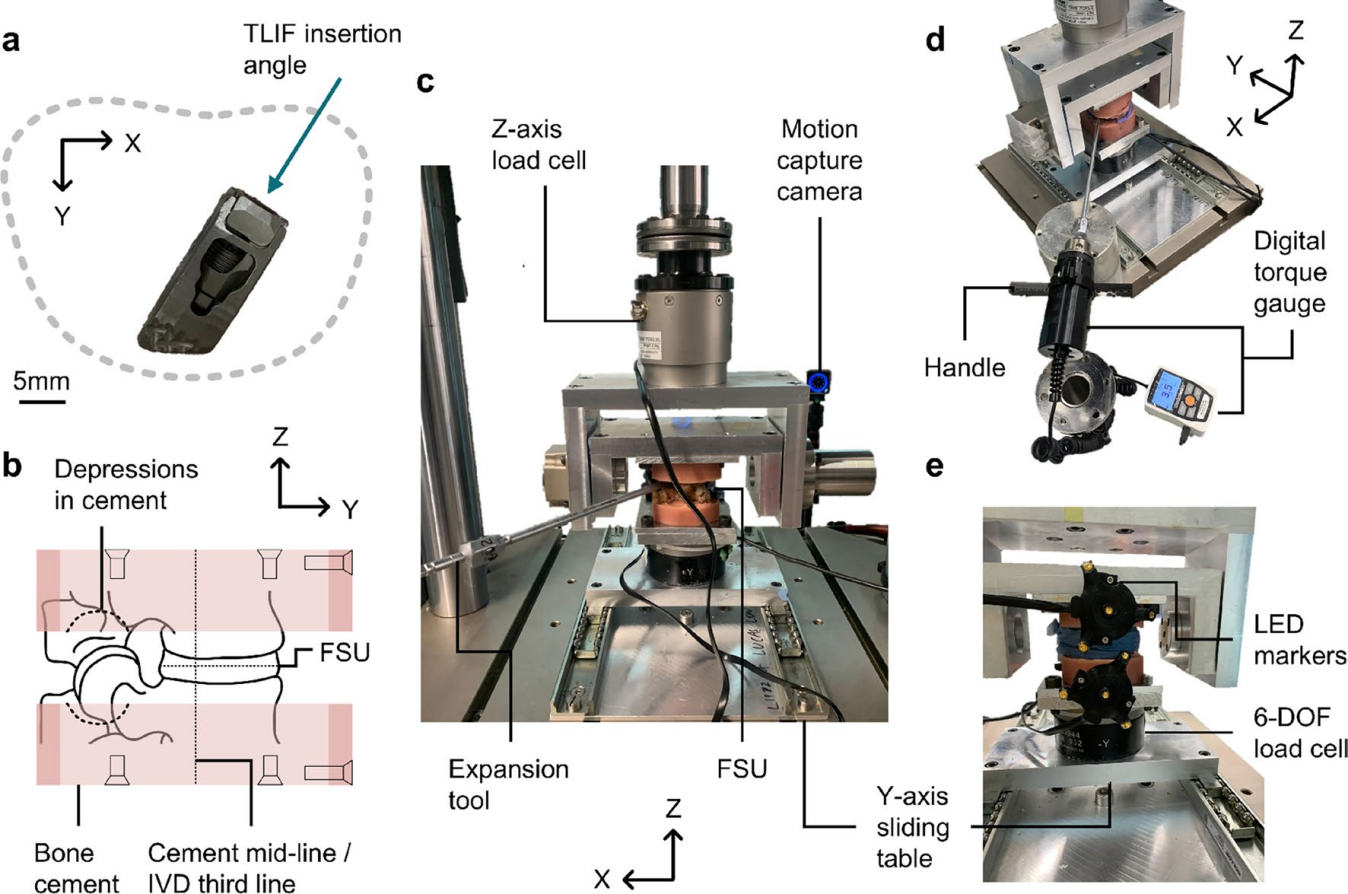
(**a**) Axial depiction of the expandable cage positioned on the endplate. (**b**) Prepared sample in bone cement. (**c**) Testing rig and sample installed into a servo-hydraulic material testing machine. The cranial fixture consists of two U-frames attached by a bearing to allow free flexion/extension through the inner U-frame, whilst the outer U-frame permits Z-axis displacement. The sample is mounted on a Y-axis sliding platform which permits anterior-posterior translation. X-axis translation, Y-axis rotation, and Z-axis rotation were restricted. (**d**) Attachment of the digital torque gauge to the expansion tool. (**e**) Clusters of LED markers fixed to the anterior cranial and caudal pots

An 8 mm distractor (Medtronic) was used to spread the IVD space in preparation for cage selection. Three titanium cage sizes (Catalyft™ interbody system, Warsaw Orthopedic, Inc., Warsaw, IN) of initial height 7 mm, 9 mm, and 11 mm, and capable of expansion by axial and lordotic change (Fig. 1a; Table 2) were available to trial, where the optimal size for the sample was selected by the surgeon.

**Table 2:** Maximum axial height and Lordotic angle changes of three expandable cage sizes (7 mm, 9 mm, 11 mm) per half-turn

Half-turn	7 mm		9 mm		11 mm	
	Axial (mm)	Lordosis (°)	Axial (mm)	Lordosis (°)	Axial (mm)	Lordosis (°)
1	5.9	0.5	9.1	0.5	11.3	0.0
2	6.4	1.0	9.2	1.0	11.5	0.0
3	7.0	2.5	9.7	2.5	11.9	2.5
4	7.6	4.0	10.1	4.0	12.3	5.0
5	8.3	6.0	10.6	6.0	12.8	7.0
6	8.9	8.0	11.0	8.0	13.3	9.0
7	9.6	10.5	11.6	10.5	13.6	11.0
8	10.2	13.0	12.1	13.0	13.9	13.0
9	10.8	14.5	12.6	15.0	14.3	14.5
10	11.3	16.0	13.0	17.0	14.6	16.0
11	11.9	18.0	13.4	18.0	15.0	18.0
12	12.4	20.0	13.7	19.0	15.3	20.0
13	12.7	21.0	14.0	20.5	15.5	21.0
14	13.0	22.0	14.2	22.0	15.6	22.0

The lumbar spines were then sectioned and potted using poly-methyl-methacrylate (PMMA) such that the mid-IVD plane was as parallel to the horizontal as possible, and the mid-coronal plane of the pot was aligned with the posterior third of the sample (Fig. 1b) [12]. Depressions in the cement were created during setting to allow physiological facet articulation (Fig. 1b). Samples were regularly sprayed with phosphate-buffered saline (PBS, 0.15 M) and, when stored, wrapped in PBS-soaked paper towel, double-bagged, and kept at −20 °C.

### Biomechanical testing protocol

Specimens were thawed in 4 °C PBS for at least 16 h prior to testing, under 100 N preload to replicate in vivo intradiscal pressures when supine [13].

Prior to testing, samples were wrapped in PBS-soaked tissue, installed into the testing apparatus attached to a servo-hydraulic materials testing machine (Instron 8874, High Wycombe, UK) (Fig. 1c-e), and subjected to an additional two hours of preload (100 N). A calibrated fluoroscopy image (resolution: ~0.09mm^2^) was captured before inserting the interbody cage as anteriorly as possible. Further fluoroscopy images verified the anterior-posterior location.

**Fig. 2:**
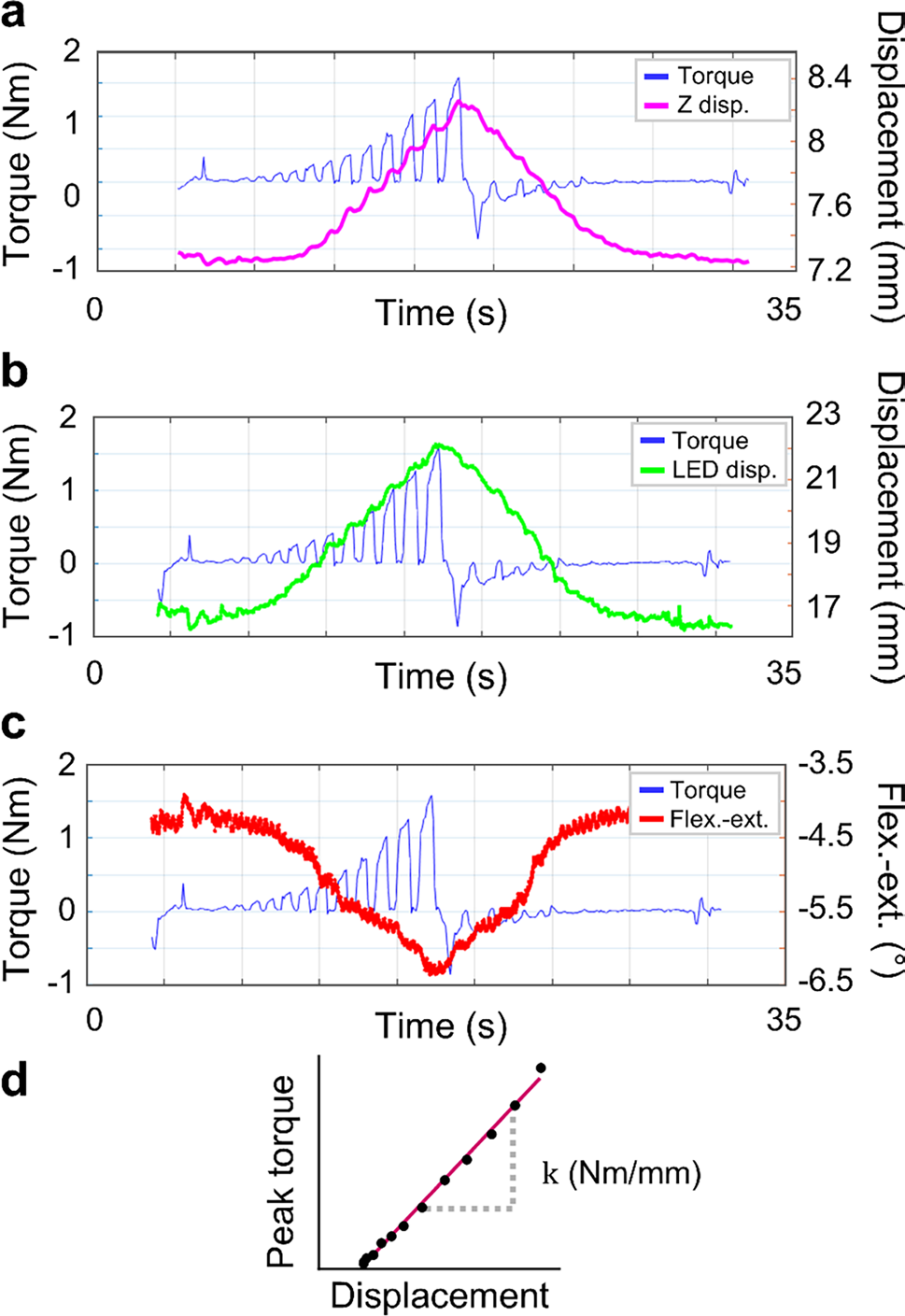
Typical torque and (**a**) transducer axial displacement, (**b**) LED marker axial displacement (both normalised to preloaded IVD height), and (**c**) flexion-extension angles during cage expansion to 14 half-turns. (**d**) Calculation of distraction stiffness (*k*) by plotting the torque peaks and corresponding displacements

A digital torque gauge (TT03-50Z, Mark-10, Germany; 5.7 Nm maximum torque, ± 0.029 Nm accuracy) was attached to the expansion tool (Fig. 1d). Under 100 N, the cage was manually expanded in half-turns until maximum expansion (14 half-turns) and collapsed four times (Fig. 2a-c), as preliminary analysis demonstrated that the fourth trial was considered repeatable due to viscoelastic IVD relaxation (Supplementary Fig. 1).

Distraction stiffness was calculated as the best linear fit of the torque-displacement data, where the displacement per half-turn was measured three ways: (1) *Axial stiffness*: To isolate axial changes in IVD height relative to the preloaded height, Z-axis displacements were measured from the servo-hydraulic transducer (Fig. 2a,d). (2) *Rotational stiffness*: To measure the IVD height accounting for lordotic changes, light emitting diode (LED) markers were attached to the top and bottom pots (Fig. 1e). Relative rigid body translations and flexion-extension of the samples were tracked using a four-camera motion capture system (Motive v2.3.7, OptiTrack, Corvallis, OR, US) (Fig. 2b,c,d). A custom-written MATLAB script calculated transformation matrices and Euler angles. (3) *Cage expansion stiffness*: To assess any variability between cage height and resultant changes to the IVD height, displacement was also recorded as the cage instantaneous maximum height (Table 2).

Fluoroscopy was used to verify that the expansion had not caused visible endplate violation. Samples were then subjected to 5000 cycles of 300–1340 N compression at 2 Hz [14], at maximum cage expansion, and under the same boundary conditions as the expansion protocol. 300 N reflected the estimated load on the cage after fixation of posterior instrumentation [15]. Since “heavy” compressive loads in the lumbar IVD can range between 1000 and 3000 N [16], two samples (#2.1 and #2.2) were used in a preliminary analysis to determine the load which resulted in subsidence within a clinically relevant range (2–4 mm [11]) after 5000 cycles. Additionally, since ~ 33% of load through the cage is tolerated by posterior instrumentation [15], 1340 N (67% of 2000 N) was chosen as the maximum load.

**Fig. 3:**
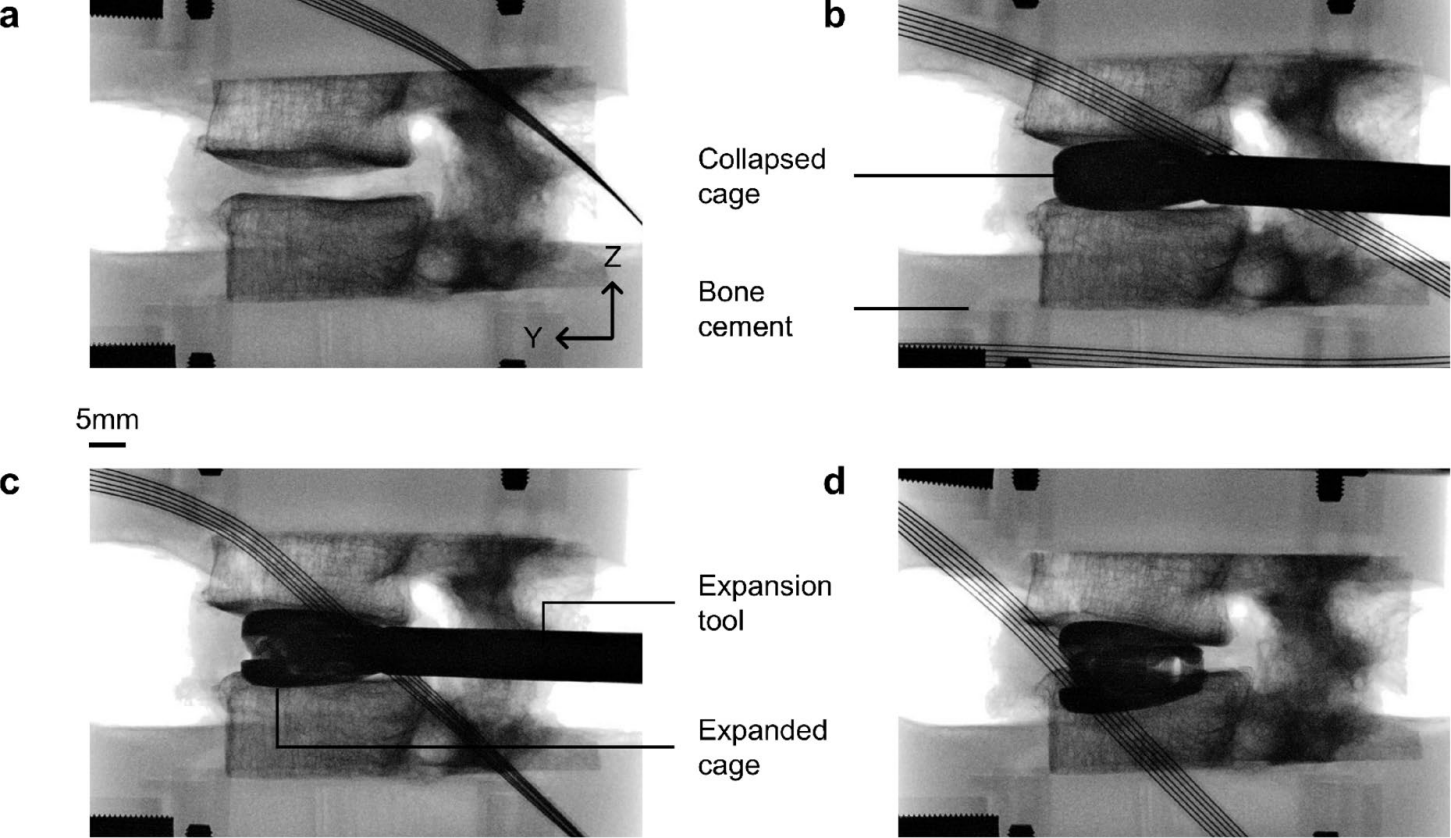
Typical sagittal fluoroscopic images (Sample #9.2 shown) acquired (**a**) after preload, (**b**) after cage installation, (**c**) after cage expansion, and (**d**) after 5000 loading cycles

Subsidence depth was calculated by subtracting the maximum displacement of the first cycle from the last cycle, in accordance with prior published work [17], and accounting for the compliance of the testing apparatus (Supplementary Methods, Online Resource). Compliance during the expansion test (100 N compression) was 0.049 mm, which was considered negligible. A final fluoroscopy image confirmed subsidence (Fig. 3).

### Radiographic variables

To assess IVD degeneration, each IVD was Pfirrmann graded [18] by two experienced raters (KR, NN) using a sagittal T2-weighted turbo-spin echo sequence (repetition time: 4540 ms, echo time: 124 ms, slice thickness: 5 mm, in-plane resolution: 0.98 mm^2^) on a 3 T MRI scanner (Magnetom Skyra, Siemens; Erlangen, Germany). Additionally, median T2 relaxation times of each IVD were calculated using a protocol described by Marinelli *et al.* [19] from a T2-weighted multi-echo sequence (repetition time: 2780 ms, echo times: 16, 32, 48, 64, 80, 96, 112, 128, and 144 ms).

To assess volumetric bone mineral density (vBMD), axial quantitative-CT images were acquired (voltage: 110 kV, current: 100 mAs, resolution: 0.98 × 0.98 × 0.60 mm) using a clinical scanner (IVIS SpectrumCT Imaging System, Caliper Life Sciences, Hopkinton, MA, US). A calcium hydroxyapatite phantom (QRM GmbH, Möhrendorf, Germany) was placed underneath specimens to convert Hounsfield units (HU) to BMD. The trabecular core (TB-vBMD) and endplate region (EP-vBMD) were segmented in Simpleware (v2024-12, Synopsys, Sunnyvale, CA, US) (Supplementary Methods, Online Resource) [20]. Voxels of low density or negative HU were assigned a value of 10 mg/cm^3^ [21]. CTs were additionally used to assess facet degeneration by two raters (KR, NN) using the Weishaupt scale [22].

Preloaded IVD height was measured from sagittal fluoroscopy images, at five equally spaced locations across the anterior-posterior IVD width. Centre point ratio (CPR) was calculated by measuring the distance between the cage centroid and the most posterior aspect of the caudal endplate, divided by the caudal endplate anterior-posterior width.

### Discectomy removal

**Fig. 4:**
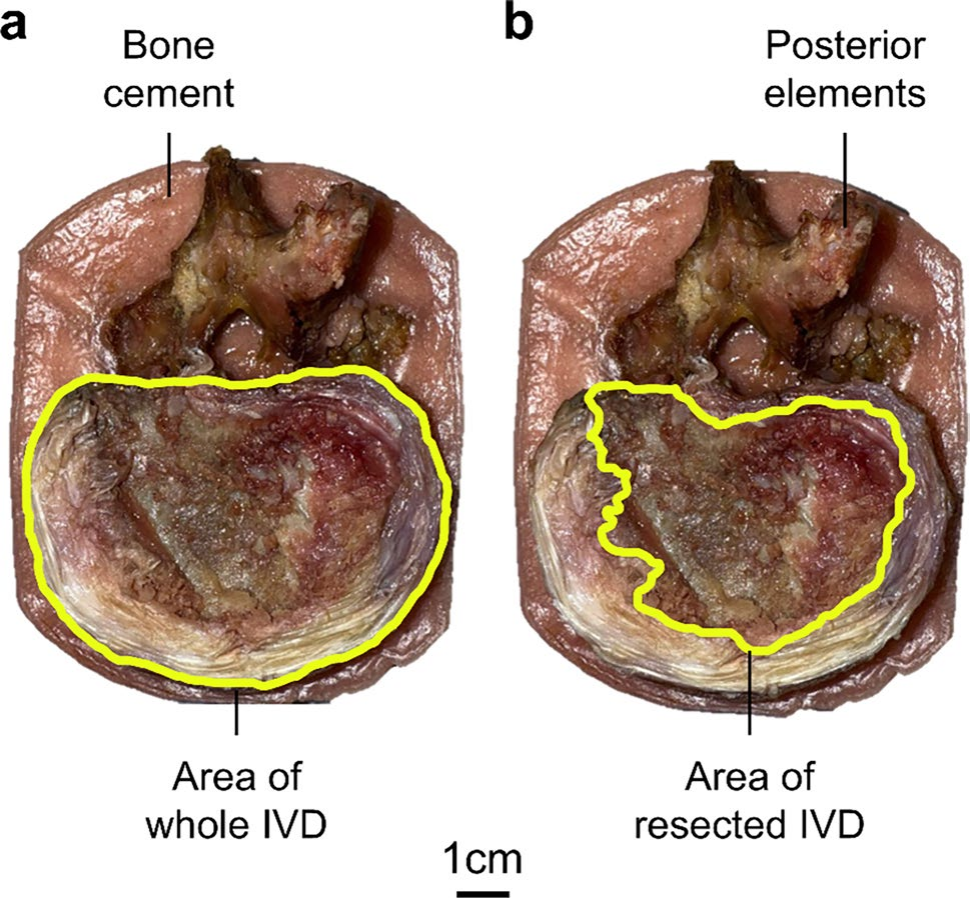
(**a**) Segmentation of the whole IVD cross-sectional area and (**b**) region of IVD resection indicated by yellow tracing (Sample #1.1 cranial half shown)

After testing, a mid-axial incision was made through the IVD, and the cranial and caudal endplates were photographed. The percentage surface area of IVD removed was calculated in ImageJ (v1.53t, Bethesda, MD, US) by dividing the segmented resected region by the whole IVD cross-sectional area (Fig. 4), averaging measurements over each endplate. Calculations were repeated twice by one blinded rater (KR) one day apart, and averaged to obtain a single percentage.

### Statistical analysis

Statistical analyses were performed in SPSS (v30.0, IBM, Chicago, IL, US) and Prism (v10.0.2, Graphpad, San Diego, CA, US). Stepwise multiple linear regression was used to predict peak torque and distraction stiffness. The variance inflation factors (VIFs) of all variables were assessed for multicollinearity, where a VIF < 10 was acceptable. To assess whether torque and stiffness were related to subsidence depth after 5000 cycles, subsidence depth was normalised to vBMD (Eq. 1) [14], and univariate linear regression was performed.

1 $$\begin{aligned} B & MD~normalised~subsidence~depth \\ & = ~\frac{{individual~vBMD}}{{mean~vBMD}}~ \cdot absolute~subsidence~depth \\ \end{aligned}$$

## Results

### Peak torque and stiffness variability between samples

During TLIF preparation, Sample #7 sustained intra-operative endplate violation and thus was excluded from subsequent analyses (Table 1).

**Fig. 5:**
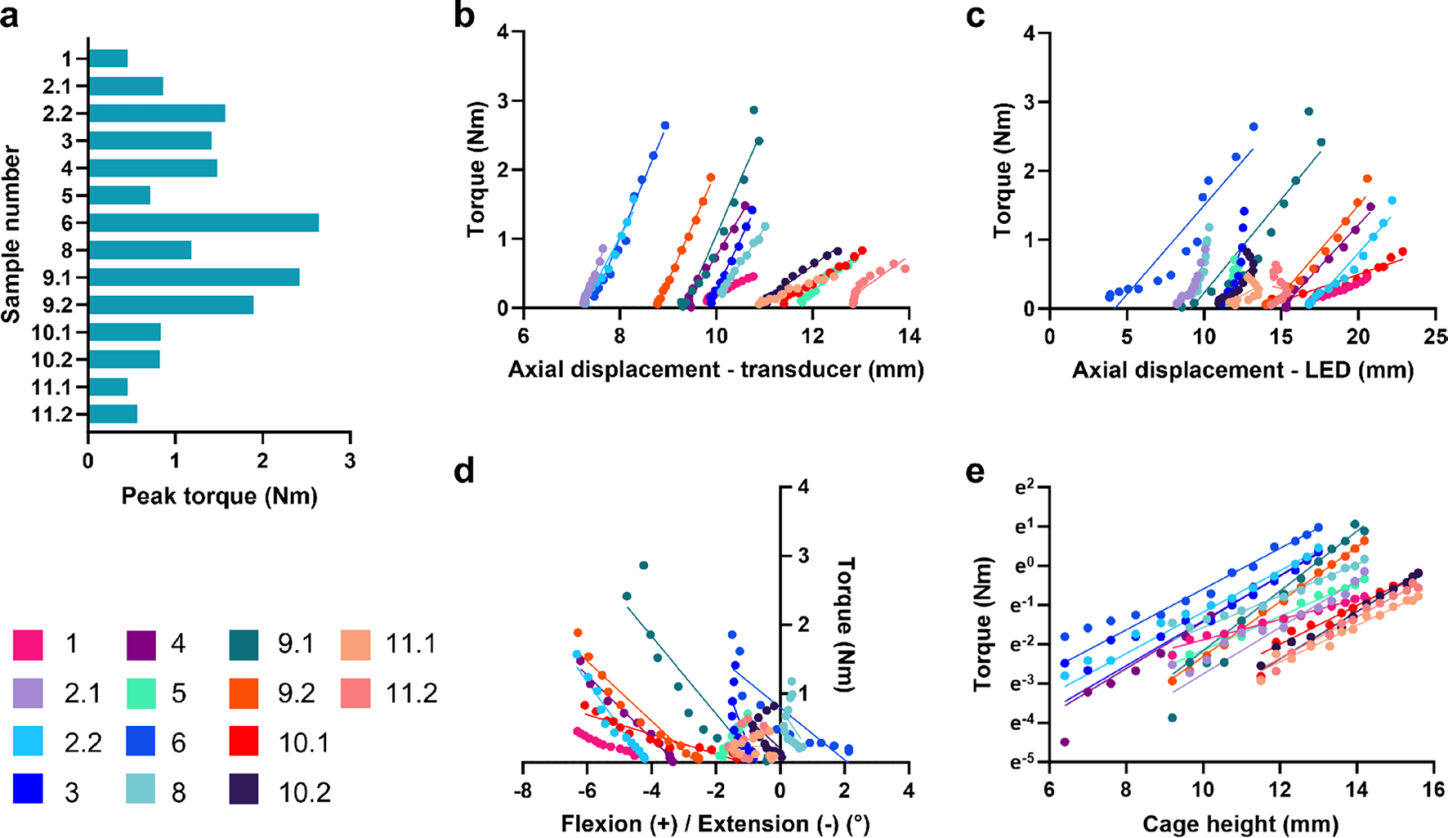
(a) Peak torque values for each sample. (**b-e**) Linear fits for (**b**) axial stiffness, (**c**) rotational stiffness, (**d**) flexion-extension angle, and (**e**) cage stiffness for individual samples (note that a natural logarithmic scale is used on the y-axis)

Across specimens, peak torque ranged between 0.455 and 2.642 Nm (Fig. 5a). Axial distraction stiffness ranged between 0.264–1.837 Nm/mm, and exhibited good linear fits (R^2^: 0.80–0.99, all *p* < 0.0001). Rotational distraction stiffness ranged between 0.077–0.766 Nm/mm (R^2^: 0.37–0.98), where one sample (#11.2) was excluded from further analysis due to an inadequate linear fit (R^2^: 0.007, *p* = 0.79). For both stiffness metrics, a linear fit was sufficient despite small non-linearities between displacement and torque in some samples (residual plots shown in Supplementary Fig. 2–4), as logarithmic transformation did not improve the linearity. However, cage height expansion and torque exhibited a non-linear relationship, so the natural logarithm was taken before calculating the fractional stiffness index per mm cage height increase (range: 0.261–0.897 mm^− 1^, R^2^: 0.94–0.99) (Fig. 5b-e).

### Prediction of subsidence

**Fig. 6:**
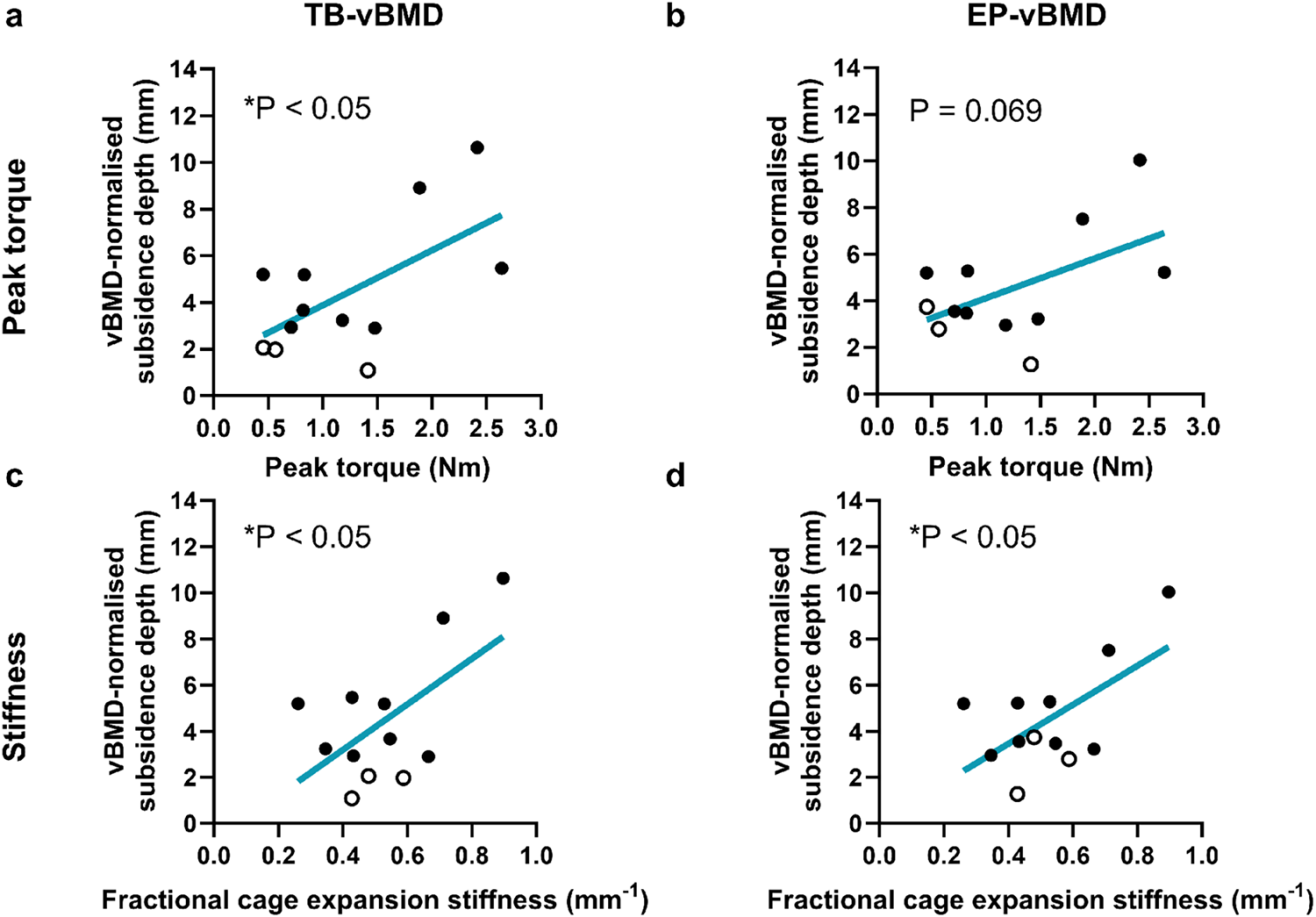
Correlations between (**a**, **b**) peak torque and (**c**, **d**) fractional cage expansion stiffness with (**a**, **c**) trabecular vBMD- (TB-vBMD) and (**b**, **d**) endplate vBMD- (EP-vBMD) normalised subsidence depth. Osteoporotic samples are included in the correlation analysis but highlighted as outlined circles for reference

Subsidence depth normalised to TB-vBMD significantly correlated with peak torque (*p* < 0.05) (Fig. 6a). Normalising to EP-vBMD weakened the positive correlation (*p* = 0.069) (Fig. 6b). However, subsidence depth normalised to both TB-vBMD and EP-vBMD significantly correlated with fractional cage expansion stiffness (*p* < 0.05), and this relationship was not dependent on the presence of osteoporotic samples (Fig. 6c, d). Regression residual plots are provided in Supplementary Fig. 5.

### Factors affecting peak torque and stiffness

Pfirrmann grade was removed prior to conducting stepwise regression, as an a priori analysis revealed a VIF > 10 without displaying any significant interactions (*p* > 0.49).

Peak torque was negatively associated with preloaded IVD height (B = −0.344, *p* < 0.001) and discectomy removal percentage (B = −0.041, *p* < 0.01) (Table 3). In other words, lower initial IVD height and less effective discectomy predicted a higher peak torque. Axial distraction stiffness was also negatively associated with preloaded IVD height (B = −0.188, *p* < 0.001), but discectomy removal percentage was not significant (*p* = 0.57). In both models, the removal of osteoporotic samples did not change statistical outcomes.

Rotational distraction stiffness was positively associated with L4-L5 segments (B = 0.312, *p* < 0.05), and negatively associated with CPR (B = −1.422, *p* < 0.05). In other words, L4-L5 segments with more posterior cage placement required more torque to distract when accounting for lordotic changes. In all models, IVD T2 relaxation time and facet degeneration had no significant influence on the peak torque or stiffness (Table 3). Models predicting fractional cage expansion stiffness were not significant. Regression residual plots are provided in Supplementary Fig. 6.

**Table 3:** Regression parameters for peak torque, axial distraction stiffness, and rotational distraction stiffness. Hyphen indicates parameter was not included in the final stepwise regression. B: unstandardised beta value; SE: standard error

	Peak torque (Nm)			Axial stiffness (Nm/mm)			Rotational stiffness (Nm/mm)		
F	18.21			19.80			6.10		
Adjusted R^2^	0.726			0.591			0.459		
Adjusted P	< 0.001			0.006			0.019		
Predictor	**B**	**SE**	**p**	**B**	**SE**	**p**	**B**	**SE**	**p**
Vertebral level (L3-L4 or L4-L5)	–	–	–	–	–	–	0.312	0.100	0.011
Preloaded disc height	−0.344	0.057	< 0.001	−0.188	0.042	< 0.001	–	–	–
Relaxation time	–	–	–	–	–	–	–	–	–
Centre point ratio	–	–	–	–	–	–	−1.422	0.630	0.048
Discectomy surface area removal %	−0.041	0.011	0.004	–	–	–	–	–	–
Facet degeneration	–	–	–	–	–	–	–	–	–
Constant	5.172	0.732	< 0.001	2.439	0.333	< 0.001	0.875	0.308	0.018

## Discussion

This study, for the first time, verified that peak torque and distraction stiffness of the IVD space were important risk factors for post-operative subsidence, and characterised their relationship with patient anatomy, surgical factors, and clinical outcome.

The variability in distraction torques (Fig. 5) may explain how haptic feedback has remained an unconvincing method to determine expansion limits in practice, particularly when considering that the degeneration status of the surrounding anatomy was not found to predict peak torque. In contrast, pre-operative IVD height was strongly associated with torque and distraction stiffness (Table 3). These findings support a prior investigation demonstrating a similar relationship between compressive stiffness and IVD height, independent from metrics representing IVD degeneration, such as AF lamellar bulging [23]. The present study suggests that the distraction stiffness of short IVDs, which clinically translates to the degree of resistive feedback to the surgeon during intra-operative expansion, can be reduced through more aggressive discectomy, but is not dependent on the hydration of the soft tissue. Given that this investigation controlled the hardware design across tests, is not currently clear how the geometry or type of expandable interbody system affects this resistance.

Peak torque and fractional cage expansion stiffness correlated with BMD-normalised subsidence depth (Fig. 6). The tensioning of the IVD space should, therefore, not be dismissed during physiological loading; at lower loads (~300 N), the cage-endplate interface may experience forces linearly related to the distraction force, and even exceed the applied preload magnitude [7], which may contribute to endplate fatigue failure upon higher loads (1000–2000 N). This finding additionally supports a prior study using bone surrogate, which demonstrated that cage-endplate contact forces directly correlated with input torque [24]. However, no correlation was found in a similar cadaveric investigation [7], thus, force at the cage-endplate interface alone may not be an appropriate metric of subsidence depth.

**Fig. 7:**
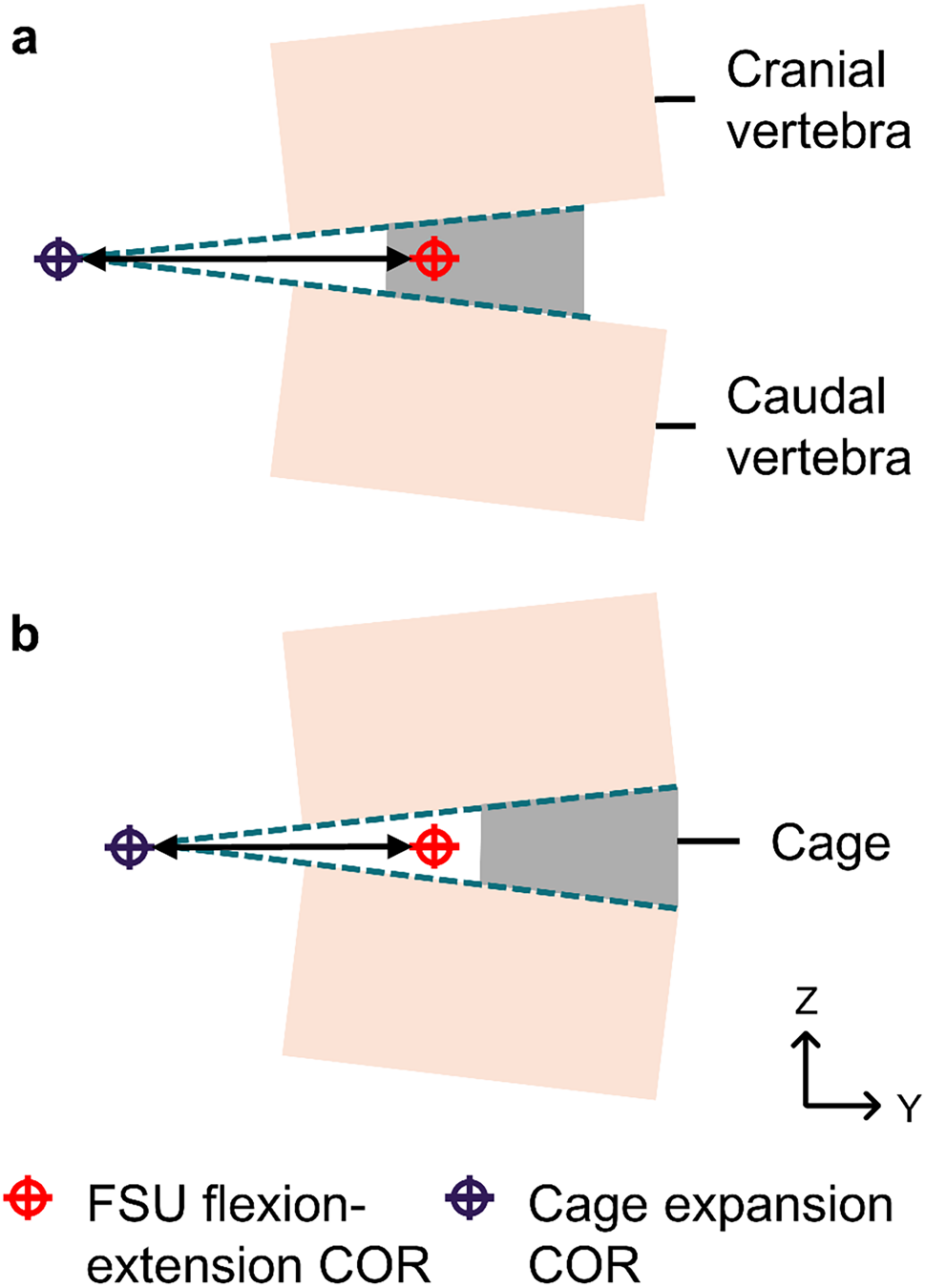
Visual representation of the repercussions to the cage expansion centre of rotation (COR) when the cage is in a (**a**) medial position *versus* (**b**) anteriorly placed. In (**a**), the COR about which the cage expands is more distal to the sample’s natural centre of rotation (estimated as 30–40% of the vertebral anterior-posterior width (Pearcy et al. [12]), than in (**b**), which is hypothesised to generate more resistance to extension

It is interesting that CPR was negatively associated with rotational distraction stiffness. This suggests that more posterior placement increases the torque required to maximise segmental lordosis. The increased resistance could be explained by the overhanging vertebra providing a counter-moment to the applied torque, combined with the cage axis of expansion located more distal to the IVD axis of rotation (Fig. 7) and a lower initial posterior IVD height relative to anterior. This reasoning supports recommendations of placing interbody cages as anteriorly as possible to optimise lordotic gain [3]. However, due to the relatively poor linear fits of LED axial displacement (and more prominently, flexion-extension angle (Fig. 5d)) with torque, caution should be taken with this interpretation.

This study has some limitations. Consistent endplate preparation across samples could not be guaranteed, which adds uncertainty to the subsidence prediction model and the validity of EP-vBMD as a covariate, although measuring EP-vBMD at the cage contact site accounted for local strength fluctuations within the endplate. Given that samples with obvious endplate breeches were excluded, and that the magnitude of endplate decortication plays an inferior role in subsidence relative to TB-vBMD [25], it is unlikely that the effect of endplate preparation would have imparted bias on the observations.

Expansion stiffness measured with axial, rotational, and cage-based displacements provided discrepant results. Fractional cage expansion stiffness being the only metric to correlate with subsidence depth demonstrates that changes in cage height within the IVD space do not necessarily translate to vertebral kinematics, where the axial and rotational distraction stiffness may be dampened by inadequate engagement of the cage with the endplate, endplate deformation, or even relative motion between the cement and vertebra. This hypothesis is reflected in the results, as the correlation between subsidence depth and axial distraction stiffness remained positive but insignificant (*p* = 0.11, Supplementary Fig. 7). However, the potential effects of endplate deformation are expected to be systematic across samples, as all stiffness metrics were independent of TB-vBMD (*p* > 0.09). Furthermore, unwanted vertebra-cement motion is likely to be minimal under compressive load, and the restriction of X-axis translation, Y-axis rotation, and Z-axis rotation during testing provided additional stabilisation to limit interfering movements, alongside representing lateral stability from missing anatomical structures that would be present in vivo (e.g., paraspinal muscles and pelvis) which would in part compensate for the unilateral facetectomy.

Additionally, the range of degeneration grades in the present study may not represent a clinical cohort. Whilst Pfirrmann 2 IVDs have minimal clinical translation, inclusion of Pfirrmann 2 IVDs in this study demonstrated the impact of increasing degeneration on the outcome measures. It is of interest to explore Pfirrmann 5 IVDs in the future; this could, however, pose challenges related to osseous growths within the IVD space and facets to give rise to endplate violation during cage insertion, as was observed in the one Pfirrmann 5 graded sample in this study.

## Conclusions

This study found high inter-subject variability in IVD distraction stiffness and achieved torque, which was directly proportionate to subsidence severity. Findings suggest that short IVDs are stiffer, and should be thoroughly prepared to minimise the torque required to achieve the desired lordosis. Since distraction stiffness is a quantifiable measure of intra-operative haptic feedback, findings from this study may contribute to the acclimatisation of digital torque drivers in expandable technology, which provide real-time readings that could be used directly to predict the patient-specific expansion threshold, or be limited to the maximum advisable torque estimated during pre-operative planning.

## Supplementary Information

Below is the link to the electronic supplementary material.

Supplementary Material 1

## Data Availability

The raw data supporting the findings of this study are available from the corresponding author upon reasonable request.
