# Supplementary material for: Intervertebral disc distraction stiffness predicts endplate subsidence following transforaminal interbody cage expansion: an ex vivo study
Source: Eur Spine J. 2026 Jan 9;35(7):3774–83. doi: 10.1007/s00586-025-09715-x (PMC13372844; doi:10.1007/s00586-025-09715-x)
Supplement: Supplementary file 1 — Supplementary Material 1 [file 586_2025_9715_MOESM1_ESM.docx]

**Supplementary material**

1. **Supplementary methods**
   1. **Quantification of trabecular and endplate volumetric bone mineral density**

In order to align the CT image such that the respective vertebral endplate was parallel to the global horizontal plane, angle measurements were made between the horizontal and a line drawn parallel to the endplate in the mid-sagittal and mid-coronal planes, and the volumetric image was resampled in the direction of these measurements.

To quantify the TB-vBMD, a cylindrical volume (cross-sectional area diameter: ~25 mm) extending from the mid-axial slice of the vertebra was segmented, leaving a ~5 mm thick gap between the segmentation volume and the superior and inferior endplates.

To quantify the EP-vBMD, a 2.5 mm thick cylindrical region was segmented underneath the estimated position of cage contact. The centre point ratio measured from fluoroscopic images was used to mark the origin of the segmentation ellipsoid along the mid-sagittal line of the vertebra. The diameter of the segmentation cross-sectional area reflected the measured cage length (20 mm). To account for topological variations in the endplates, the whole vertebra was segmented using an automated function, which was then intersected with the 2.5 mm cylindrical volume using Boolean operations.

- 1. **Calculation of compliance in testing apparatus**

The testing apparatus (Y-axis sliding table, 6-DOF load cell, double U-frame fixture and 1-axis load cell) was subjected to four compressive loads in succession (800 N, 1200 N, 1600 N, 2000 N) and the displacement measured by the servo-hydraulic displacement transducer was recorded (Fig.1a). A non-linear equation was used to fit the data and interpolate the force-displacement profile. The interpolated compliance values, normalised to load magnitude, were subtracted from all displacement datapoints in the cyclic test data (Fig.1b).

| 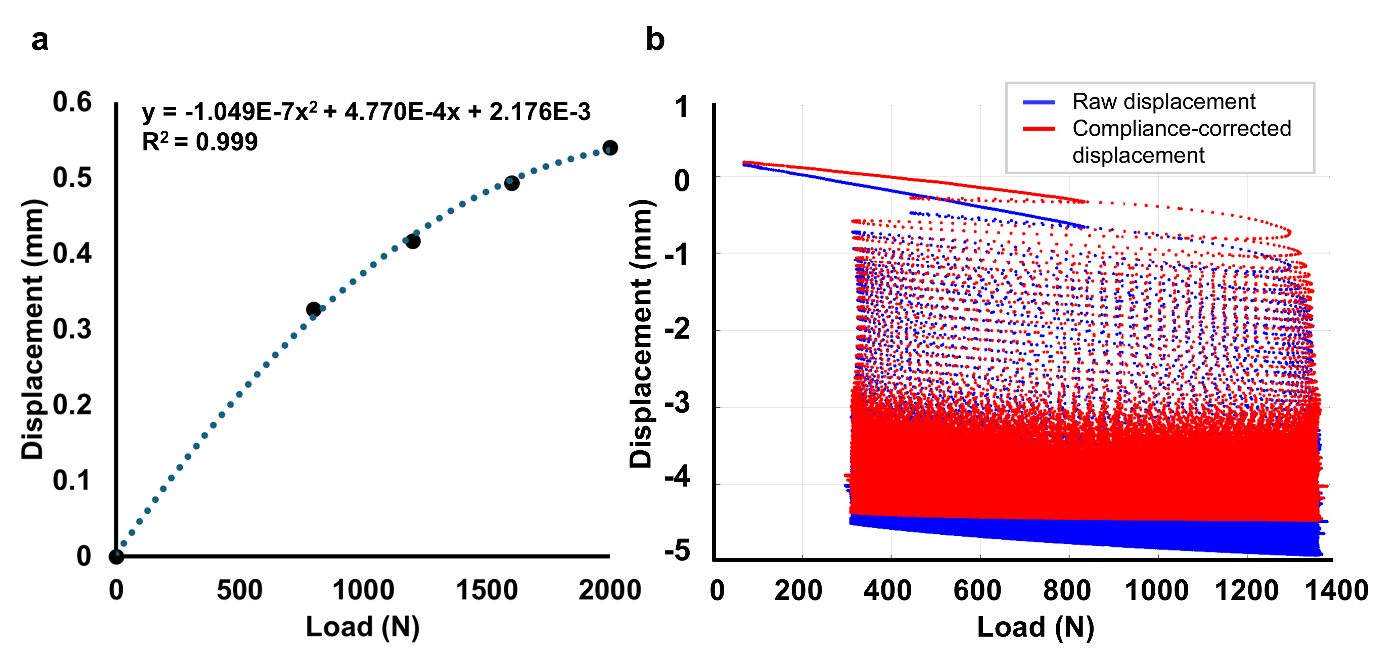 |
| --- |
| **Fig.1** (a) Compliance curve and (b) compliance-normalised force-displacement data of one sample (#6) during cyclic testing |

1. **Supplementary figures**

| 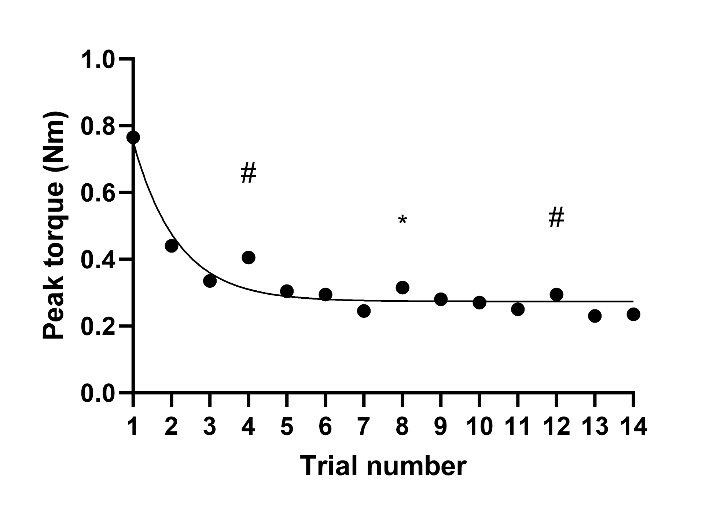 |
| --- |
| **Supplementary Fig.1** Preliminary evaluation of achieved peak torque after 14 successive trials of expanding and collapsing the cage. Small fluctuations in peak torque can be attributed to short periods of IVD recovery between trials (#) and the removal and re-insertion of the interbody cage (*) |

| 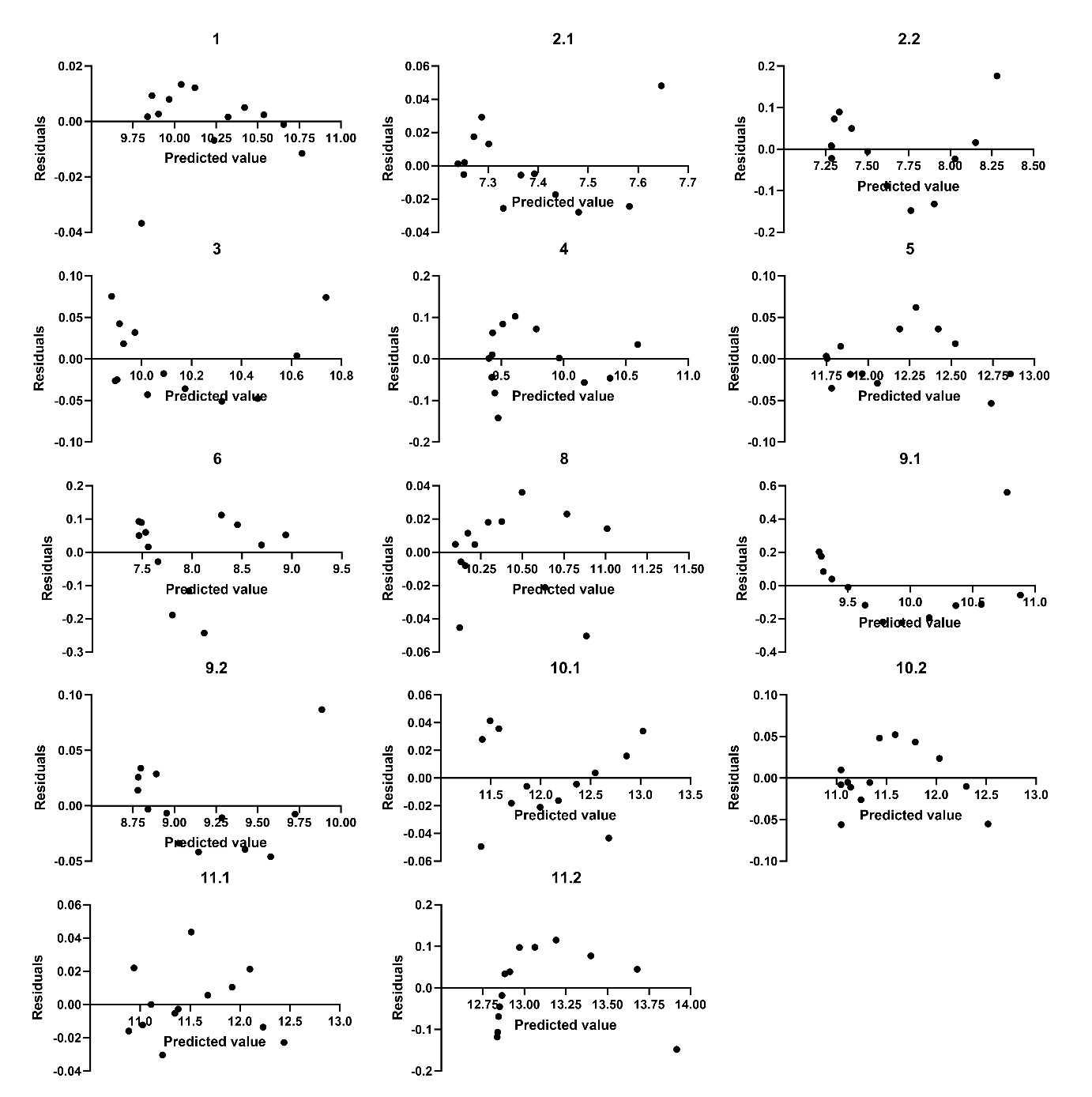 |
| --- |
| **Supplementary Fig.2** Unstandardised residual plots for axial distraction stiffness regression fits. Graphs are labelled with their respective sample number |

| 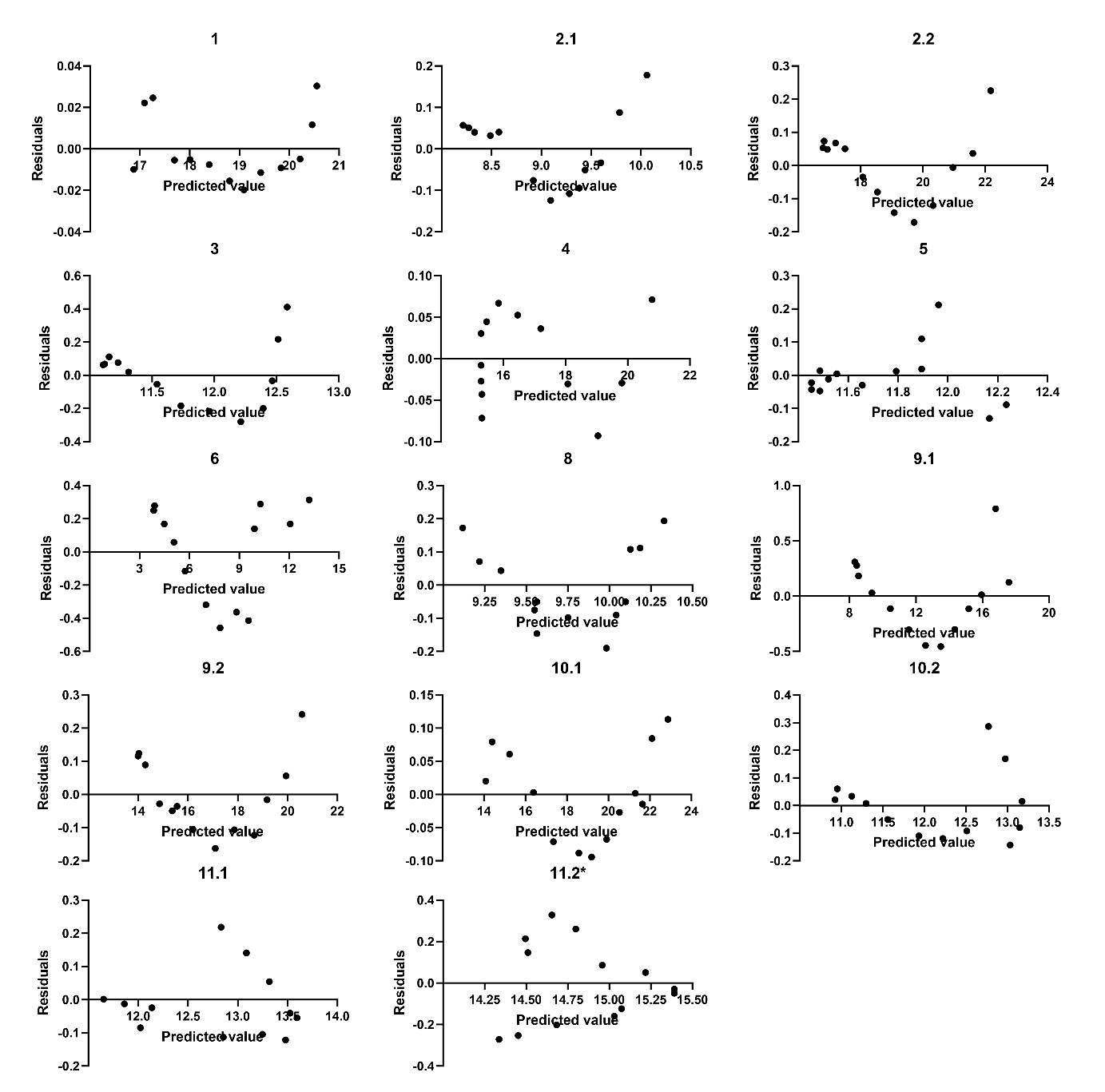 |
| --- |
| **Supplementary Fig.3** Unstandardised residual plots for rotational distraction stiffness regression fits. Graphs are labelled with their respective sample number. Asterisk denotes samples which were excluded due to insignificant linear fits |

| 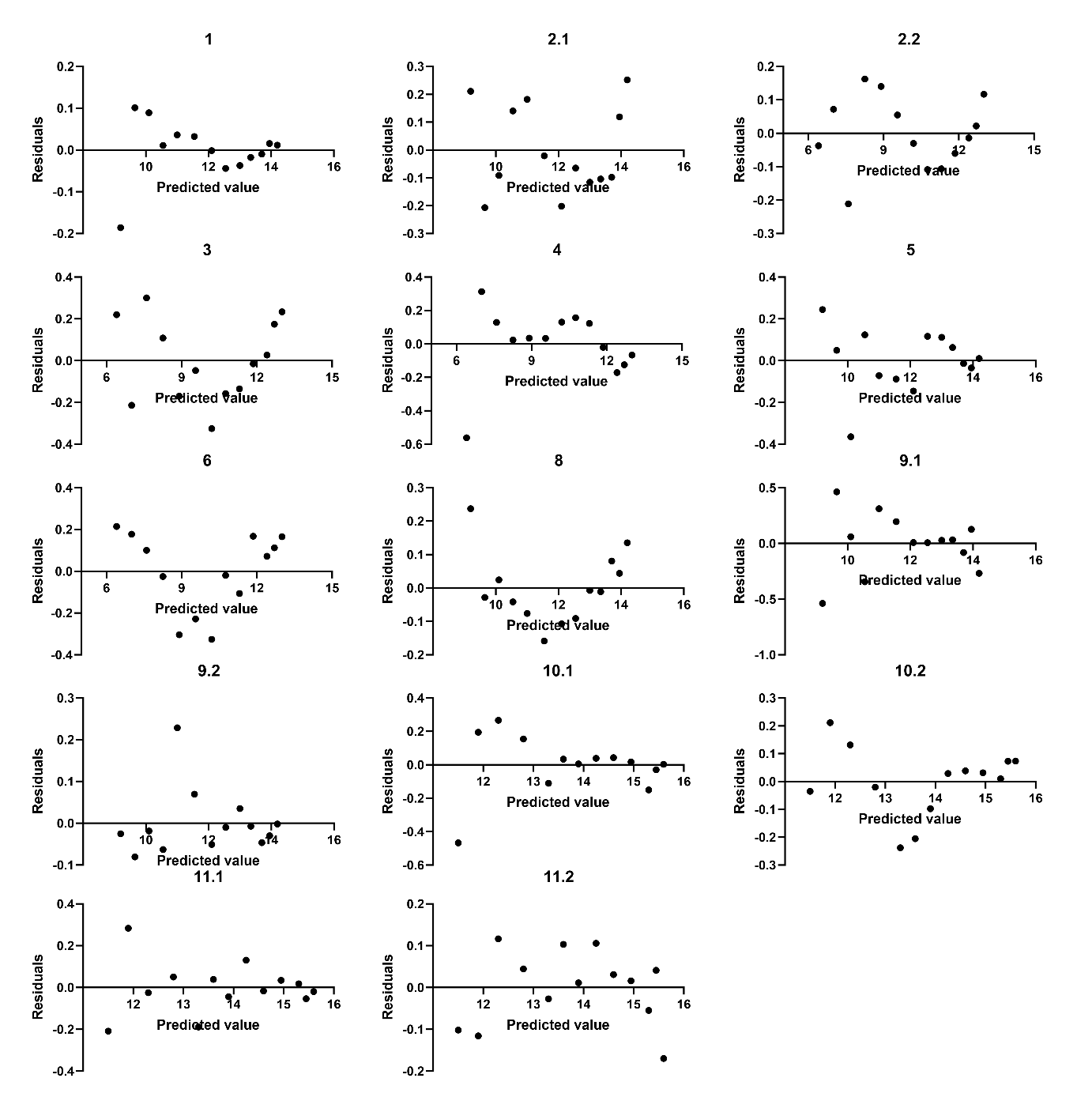 |
| --- |
| **Supplementary Fig.4** Unstandardised residual plots for cage height expansion regression fits. Graphs are labelled with their respective sample number |

| 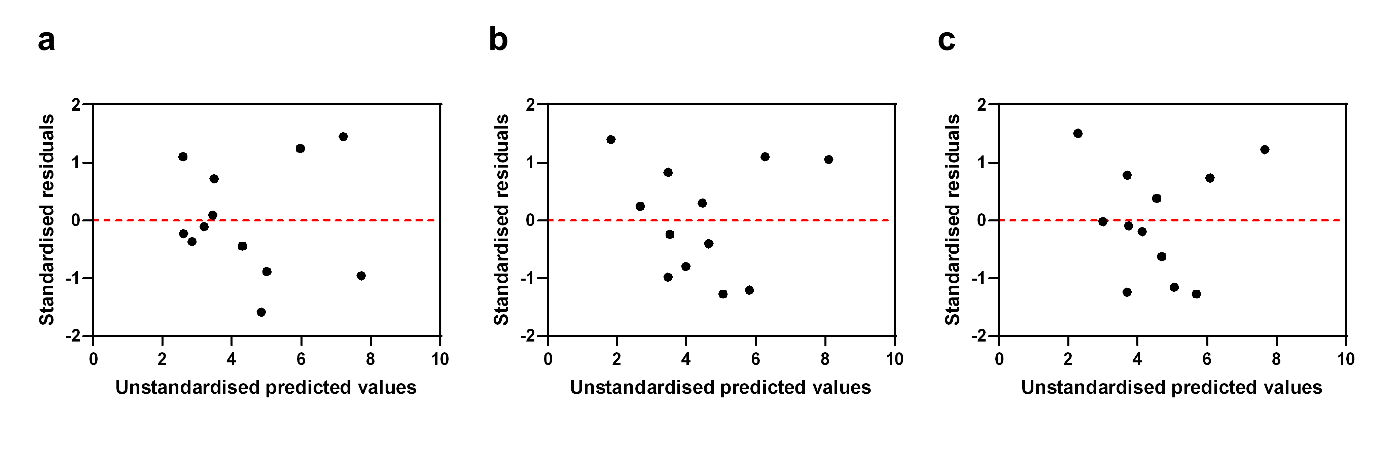 |
| --- |
| **Supplementary Fig.5** Standardised residual plots for univariate regression models predicting (a) TB-vBMD normalised subsidence using peak torque, (b) TB-vBMD normalised subsidence using fractional cage expansion stiffness, and (c) EP-vBMD normalised stiffness using fractional cage expansion stiffness |

| 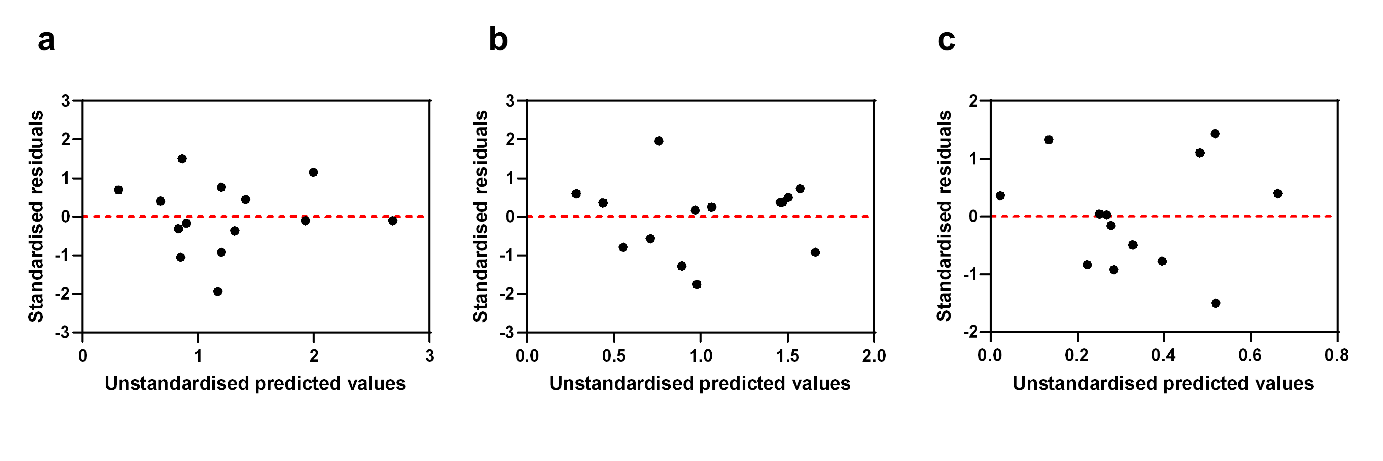 |
| --- |
| **Supplementary Fig.6** Standardised residual plots for multivariate regression models predicting (a) peak torque, (b) axial distraction stiffness, and (c) rotational distraction stiffness |

| 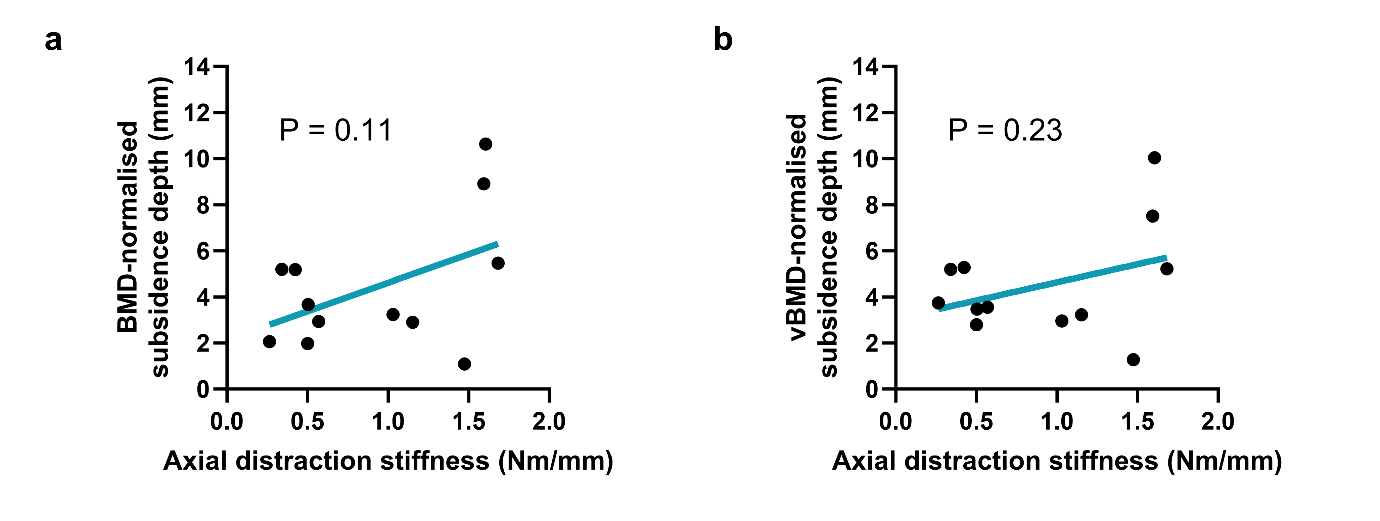 |
| --- |
| **Supplementary Fig.7** Relationship between (a) trabecular and (b) endplate vBMD normalised subsidence depth and axial distraction stiffness |
